# Accessibility of general practitioners and selected specialist physicians by car and by public transport in a rural region of Germany

**DOI:** 10.1186/s12913-016-1839-y

**Published:** 2016-10-19

**Authors:** Ulrike Stentzel, Jens Piegsa, Daniel Fredrich, Wolfgang Hoffmann, Neeltje van den Berg

**Affiliations:** Department Epidemiology of Health Care and Community Health, Institute for Community Medicine, University Medicine Greifswald, Ellernholzstrasse 1-2, 17487 Greifswald, Germany

**Keywords:** Accessibility, Car travel, Public transport, GIS, Network analysis, Health care providers, General practitioners

## Abstract

**Background:**

The accessibility of medical care facilities in sparsely populated rural regions is relevant especially for elderly people which often represent a large segment of the population in such regions. Elderly people have higher morbidity risks and a higher demand for medical care. Although travelling with private cars is the dominating traffic mode in rural regions, accessibility by public transport is increasingly important especially because of limited mobility of elderly people. The aim of this study was to determine accessibility both by car and public transport to general practitioners (GP) and selected specialist physicians for a whole region and to detect areas with poor to no access in the county Vorpommern-Greifswald, which is a rural and sparsely populated region in the very northeast of Germany.

**Methods:**

Accessibility of medical care facilities by car was calculated on the basis of a network analysis within a geographic information system (GIS) with routable street data. Accessibility by public transport was calculated using GIS and a network analysis based on the implementation of Dijkstra’s algorithm.

**Results:**

The travelling time to general practitioners (GP) by car in the study region ranges from 0.1 to 22.9 min. This is a significant difference compared to other physician groups. Traveling times to specialist physicians are 0.4 to 42.9 min. A minority of 80 % of the inhabitants reach the specialist physicians within 20 min. The accessibility of specialist physicians by public transport is poor. The travel time (round trip) to GPs averages 99.3 min, to internists 143.0, to ophthalmologists 129.3 and to urologists 159.9 min. These differences were significant. Assumed was a one hour appointment on a Tuesday at 11 am. 8,973 inhabitants (3.8 %) have no connection to a GP by public transport. 15,455 inhabitants (6.5 %) have no connection to specialist internists.

**Conclusions:**

Good accessibility by public transport is not a question of distance but of transport connections. GIS analyses can detect areas with imminent or manifest deficits in the accessibility of health care providers. Accessibility analyses should be established instruments in planning issues.

## Background

Rural areas with a low population density are characterized by sparsely distributed health care providers and facilities. Catchment areas of physician practices and hospitals are large with long travel distances for patients in many cases. Accessibility is regarded as the ease to reach destinations or activity sites [1]. Equity of access to health care providers includes the possibility to overcome spatial distances. Beyond the range of walking distances, people have to use some kind of transportation, e.g., a private car or public transport. Private cars are dominant over public transport, particularly in rural regions. However, not every citizen has access to a car. In the western part of Germany 72 % of the inhabitants have access to a car. In the eastern federal states this proportion is only 64 % [2]. About 60 % of younger single households have a car available. 80 % of two-person-households of middle age and 76 % of older two-person-households have a car. However, in only 50 % of elderly single households is a car available [2]. Citizens and households without access to a car are dependent on public transport or car ride sharing usually with friends, relatives or neighbors.

The study region is the county Vorpommern-Greifswald (3,927 km^2^, part of the federal state of Mecklenburg-Western Pomerania in the northeast of Germany). It is a rural and sparsely populated county with an average of 69 inhabitants/km^2^ [3]. The regional population is both declining and aging. Until 2030, the proportion of the population aged 60 years and older will increase by 21 % [4]. With older age, the morbidity risk and the demand for medical care rise [5–8]. General practitioners (GP) have high patient numbers and contacts in the elderly population. Of the specialist physicians in private practices, internists, ophthalmologists and urologists have the highest utilization rates in the population [9]. Increasing age is associated with increasing limitations in mobility [10, 11]. A cross-sectional survey for the study region assessed the proportion of older people holding driving licenses, having a car and preferred modes of transport [12]. In total 63 % of people aged ≥ 60 years had a driver’s license (88 % of the men and 43 % of the women). People living alone less often had a car than people living with more persons in a household (31 vs. 66 %) [12]. 70 % of the men and 19 % of the women drove the car themselves. The proportion of people who stopped driving a car due to health restrictions increased with increasing age (60 to 69 years 3 %, 70 to 79 years 8 % and 80 years and older 23 %). Public transport could be an alternative to stay mobile also in higher age. Especially the oldest old have high utilization rates and a limited mobility in many cases. This age group is presently the subpopulation with the fastest growth in Mecklenburg-Western Pomerania.

The evaluation of the accessibility of health care providers and facilities actually involves two perspectives: accessibility by car and accessibility by public transport. The accessibility by car is well known and recognized. However, the determination of the accessibility by public transport is considerably more complex and methodologically challenging. In this field only a handful of articles have been published [13–18]. All of them developed different designs to handle the complexity (identifying routes, considering footpaths and speeds, distances to stops and time tables). Lovett et al. identified bus routes where there was at least one round trip journey every weekday within a daytime to GP surgeries for the region East Anglia in the United Kingdom. Average travel times for residents in each ward were analyzed [16]. Liu and Zhu developed an “Accessibility Analyst” as an extension for a geographic information system (GIS) to support analysis in urban transportation planning. This tool provides a set of accessibility measures, including Spatial Analyst, Network Analyst, 3D Analyst and Patch Analyst [15]. Benenson et al. developed a GIS tool for the ArcGIS software called “Urban.Access” for the evaluation of accessibility that can be used in metropolitan areas throughout the world. This tool estimates car-based and transit-based accessibility values on the basis of a network and a location-based measure of accessibility. It works with a layer of network compatible roads, a layer of transit stops and roads, a layer of transit departure and arrival times and optional with a layer of land use and origin/destinations [13]. Tribby and Zandbergen as well as Djurhuus et al. used multimodal networks to evaluate accessibility. Tribby and Zandbergen realized a model to measure accessibility using a multimodal model that determined travel time considering walking times, waiting times, travel times using different transport modes and transfer times between routes for urban downtown Albuquerque, New Mexico [18]. Djurhuus et al. combined road network data and self-reported travel times and distances to work or study by walking or cycling and by public transport in a multimodal network analysis to examine the association between individual public transport accessibility and self-reported active commuting in the capital region of Denmark [14]. Also for urban settings (the capital region of Finland) compared Salonen and Toivonen accessibility by car and by public transport using three approaches: a simple, an intermediate and an advanced model, differentiated by an increasing number of included parameters. The advanced model included walking times and times needed to find parking spaces (accessibility by car) and walking and waiting times using public transport. Public transport travel times were calculated based on average speeds, derived from route lengths and approximate route drive-through-times [17].

In brief, some of these approaches works with simplifications and estimations. Some use multimodal networks, consisting of pedestrian network and public transport network, including bus routes and time tables. Those are mostly looking on urban districts or regions. Either their study regions are relatively small in its dimensions as well as in its variety of different transport companies and transport systems or they can use only one data source with uniformly structured data. The transfer between different transportation lines or systems was often unconsidered or included as estimations. The purpose of this article is to analyze the accessibility of health care providers and medical facilities both by car and by public transport for a whole rural region. In the study region, 17 public transport-companies operated with 17 different time table data formats. Also the transfers were considered, based on distances and timetables. GP-practices, internists, ophthalmologists and urologists were selected as examples for medical care facilities. Because travel time is more decisive for people than travelled distance [16, 18] the focus is on the time that is needed to reach the nearest physician. The main goal of this analysis is to identify areas with poor accessibility and to quantify the respective size of the affected population. This prompts the following research questions:

- How is the accessibility of GP, internists, ophthalmologists and urologists by car?
- How is the accessibility of these physician groups by public transport?
- Are there any differences between the physician groups?

## Methods

In this descriptive study, both accessibility analyses were realized with a geographic network-analysis, based on graph theory [19]. Traffic takes place on roads. A road network is a network with edges (roads) and vertices (crossings) in the sense of the graph theory. A graph consists of a set of nodes or vertices and a set of existing connections between these nodes, the edges [20]. Edges are described by a direction and a cost. The simplest network analysis is to determine the cheapest route from node A to node B, which are connected to each other via a path of edges. The classical algorithm to calculate routes is Dijkstra’s algorithm [21]. From a starting point (origin) this algorithm searches along directed edges from node to node the fastest or shortest route to the destination node.

The geographic information system (GIS) ArcGIS 10.0 (ESRI, Redlands, USA) provides a special toolbox to perform network analysis, the Network Analyst. The tools in this toolbox work with an implementation of Dijkstra’s algorithm.

The county Vorpommern-Greifswald has an external border to the Baltic Sea in the North and to Poland in the West, internal borders to other counties in the federal state of Mecklenburg-Western Pomerania and a border to the federal state of Brandenburg in the south. People rarely cross the external border to visit a doctor, however, they do cross internal borders; both to other counties and to other federal states. To take this into account, a 15 km buffer zone was defined around the study region to cover adjacent regions within the federal state of Mecklenburg-Western Pomerania. All relevant medical providers within the buffer were considered in the analyses. It was not possible to take into account a similar buffer for the federal state Brandenburg, because public transport data were not available.

Population data at the municipality level were retrieved from the Central Information Register (ZIR) that records the population data of Mecklenburg-Western Pomerania at a daily basis (data retrieval: 5^th^ September 2012). These data were used to determine the affected population. The population data were merged to the district level (*N* = 593 districts). Population data were available for 465 districts; some smaller districts had to be aggregated.

Location data of the physicians of the county were derived from the database of the Association of Statutory Health Insurance Physicians Mecklenburg-Western Pomerania. The addresses were geocoded in ArcGIS 10.0.

### Accessibility by car

The analysis of the accessibility by car in ArcGIS required digital routable street data and geocoded address data of the medical providers. Routable data contains information for every road section about speed limits, distances, directions (e.g., one-way streets), turn restrictions but also house numbers and postal codes for each side of the street.

For this network analysis, we used NavStreet Data from NAVTEQ and the geographical data of the Federal Agency for Cartography and Geodesy as at 2012 (“DeutschlandPlus”, Logiball, Herne).

The center points of the districts were used as origins for the trips to the medical providers. To determine zones of equal travel times to the physician practices (in 5 min distance-categories) the Network Analyst tool “New Service Area” was used to determine travel time to the practice in minutes. The exact distance values (in minutes as well as in meters) from the center points of the districts to the physician practices were calculated with the Network Analyst tool “OD cost matrix”. These values were used to calculate average distance, standard deviation and maximum distance.

### Accessibility by public transport

To determine the accessibility of physician practices by public transport, bus and train time tables (made available by the regional transport companies) were used. The geographical coordinates of bus and train stops were actively collected by study staff using a GPS device.

The travel times from each district center point to the physician practices were calculated in three steps:

1. Calculation of the footpath distances from the district center point to the three nearest bus and/or train stops;
2. Calculation of the footpath distances from the origin bus/train stop to the destination bus/train stop for vehicle changes;
3. Calculation of the footpath distances from the destination bus/train stop to the physician practice using the ArcGIS OD cost matrix tool.

All three calculations were made with the ArcGIS OD cost matrix tool. The footpath distances and the bus and train timetables are essential for the calculation of the overall public transport travel time that is made with a self-developed software based on Dijkstra’s algorithm.

The results of the calculation were visualized on a map with ArcGIS using Thiessen polygons. A Thiessen polygon defines an area of influence around its sample point, so that any location inside the polygon is closer to that point than any of the other sample points [22].

The calculation is based on some assumptions: the appointment in the physician practice is on a Tuesday at 11 am out of school holidays (the public transport is mainly organized according to school time requirements [23]). The patient starts his travel earliest at 7 am and should be back home latest by midnight. The speed of pedestrians is commonly assumed with 1 m per second which corresponds to 3.6 km per hour [24]. The focus of this study is on elderly people, who have a lower walking speed [25]. We assumed a walking speed for this group of 1.8 km per hour. The following maximum footpaths were defined:

- Up to 1,000 m from the district center point to the physician practice (in this case, we assumed the patient will not walk the distance and use public transport);
- Up to 1,000 m from the district center point to the three nearest bus and train stops;
- Up to 500 m from the destination bus/train stop to the physician;
- Up to 250 m between bus/train stops for bus/train changes.

In the calculation, the three nearest bus and train stops were considered because the nearest bus or train stop must not necessarily provide the fastest connection. The return trip is defined to start latest at 12 am.

For both calculations the duration of the doctor’s appointment is not included in the travel time. Also not considered were traffic jams, rush hours, construction sites or the search for parking lots.

The differences between the accessibility of the single physician groups were analyzed using the Kruskal-Wallis test followed by a Tukey correction to examine which means differ significantly from each other. The calculations were made with SAS 9.3 © 2002–2010 (by SAS Institute Inc., Cary, NC, USA).

## Results

### Results of accessibility by car

**Fig. 1:**
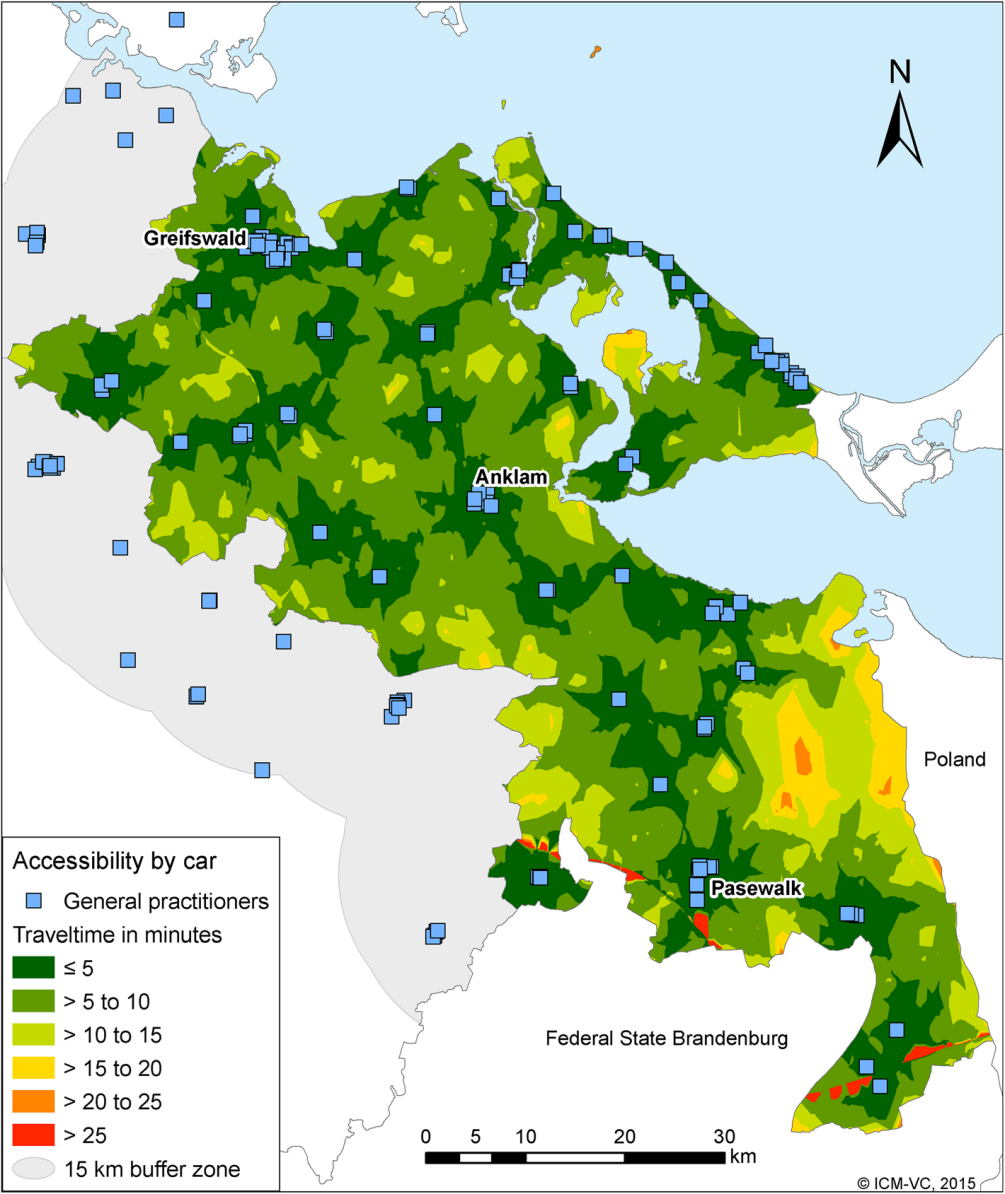
Accessibility of GP by car

Figure 1 shows zones of equal travel time ranges (one-way travel time in minutes) by car to the GP-practices in the county Vorpommern-Greifswald. The physicians are well distributed in the region; the travel times are usually low. The GP-practices are accessible from almost every municipality in the region within 15 min. The yellow and orange areas in this map represent areas with only few inhabitants. The red line-shaped areas in the south are areas next to the motorway. Motorways are massive barriers that can be crossed in only a few places.

38.6 % of the districts (representing 80.2 % of all inhabitants in the region) are located in the zone under 5 min travel time, (Table 1).

**Table 1:** Travel time to the nearest GP by car with the proportions of affected districts and inhabitants

Time (minutes)	Number of districts	Number of inhabitants	Proportion of districts	Proportion of inhabitants
<5	183	191,662	39	80
5 to < 10	235	41,486	51	17
10 to < 15	37	4,918	8	2
15 to < 20	8	1,032	2	0
≥20	1	4	0	0
Total	464	239,102	100	99^a^

^a^The sum of the percentages is 99 % because of rounding

**Fig. 2:**
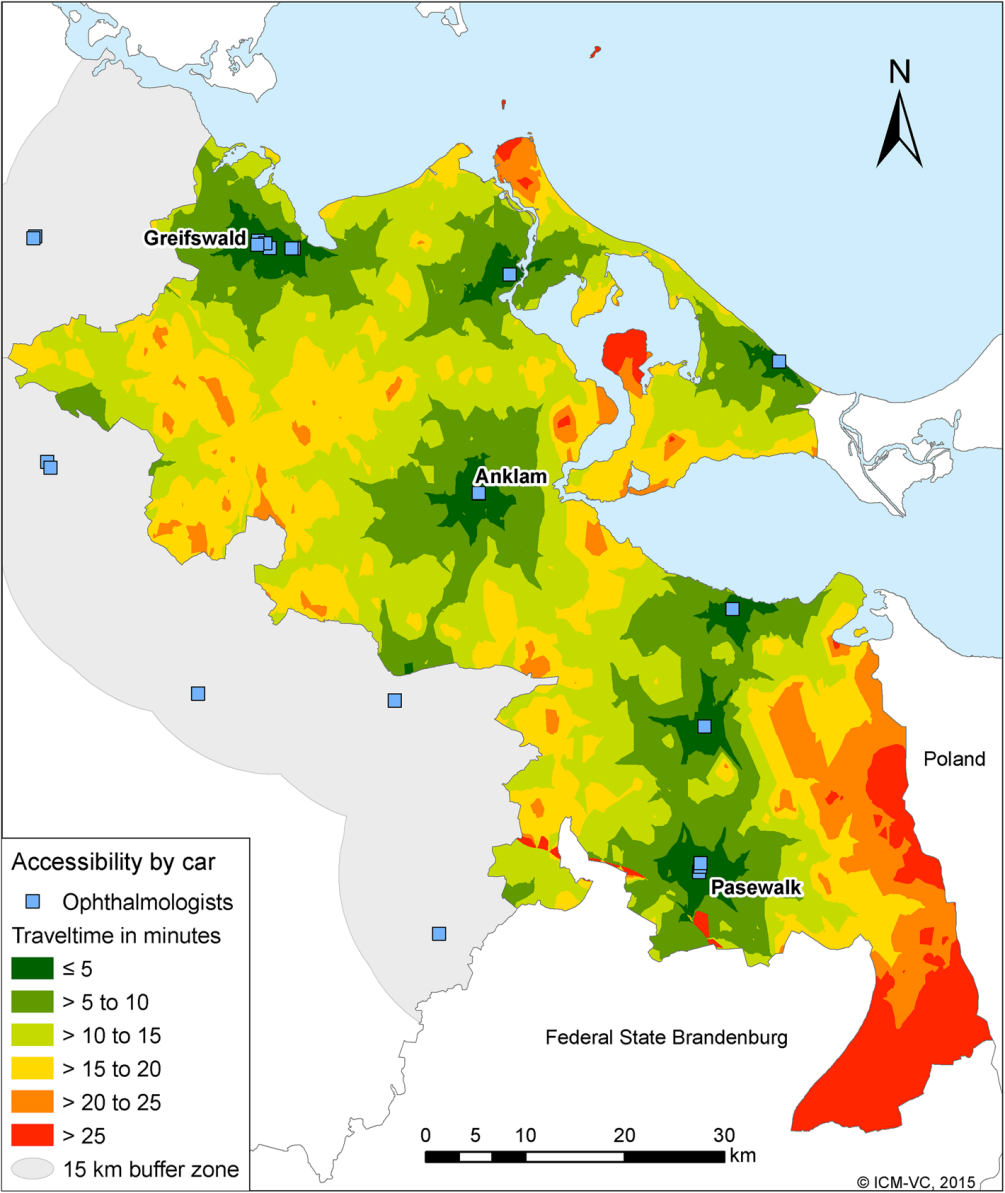
Accessibility of ophthalmologists by car

Figure 2 shows the accessibility of ophthalmologists by car as an example for a medical specialist. Overall, travelling times to medical specialists are higher compared to GPs, because ophthalmological practices are mostly located in larger towns. Only 6.4 % of the districts with 47.2 % of the inhabitants have travel times less than 5 min. More than 30 % of the inhabitants need 10 to 20 min to reach the next ophthalmologist, about 3.5 % of the inhabitants need more than 20 min. The situation is similar for the accessibility of specialist internists and urologists. A ride to the next urologist takes more than 20 min for 19.7 % of the male inhabitants.

Table 2 shows for all 4 considered physician groups the mean and maximum travel time in minutes and the standard deviation. It is apparent that the maximum travel times are outliers and that most travel time durations are near the mean. As expected the average mean travel time to GPs is lower than to the specialist physicians.

**Table 2:** Car travel distances in minutes and in kilometers to GPs, internists, ophthalmologists and urologists

Physician group	Distance	Mean	Standard deviation	Maximum
General practitioner	Minutes	6.0	3.2	22.9
	Kilometers	6.0	3.4	19.7
Ophthalmologist	Minutes	12.8	6.1	37.6
	Kilometers	14.2	7.6	46.5
Specialist internist	Minutes	13.8	6.6	37.2
	Kilometers	16.0	8.3	50.0
Urologists	Minutes	15.7	7.7	42.9
	Kilometers	17.4	9.6	48.2

The analysis of variance using Kruskal-Wallis showed that the differences in mean of the accessibility of the four physician groups by car are significant for travel time (*p* < .0001) as well as for travel distance (*p* < .0001). The subsequent Tukey correction showed that all four groups differ significantly.

### Results of accessibility by public transport

**Fig. 3:**
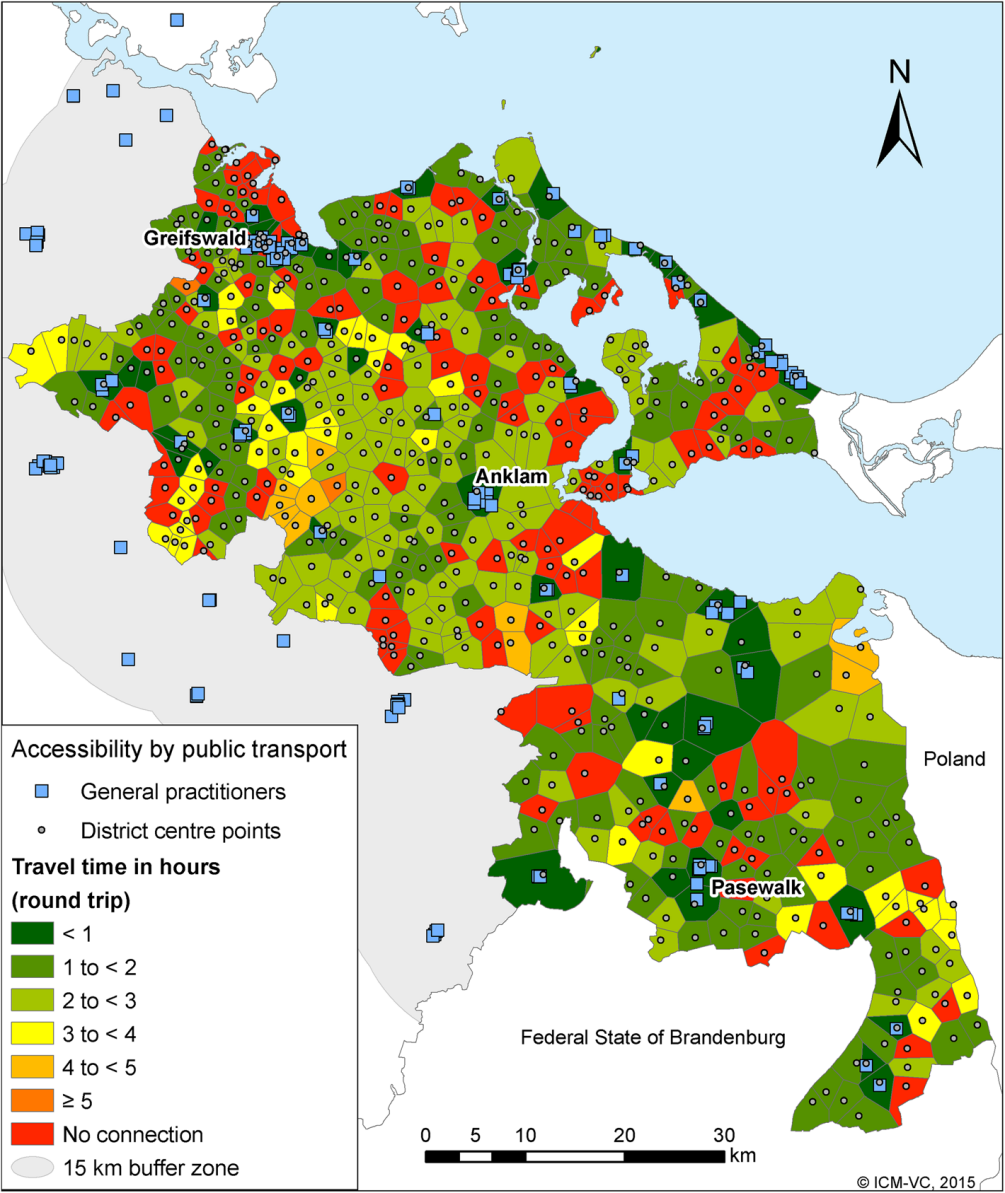
Accessibility of GP by public transport

**Fig. 4:**
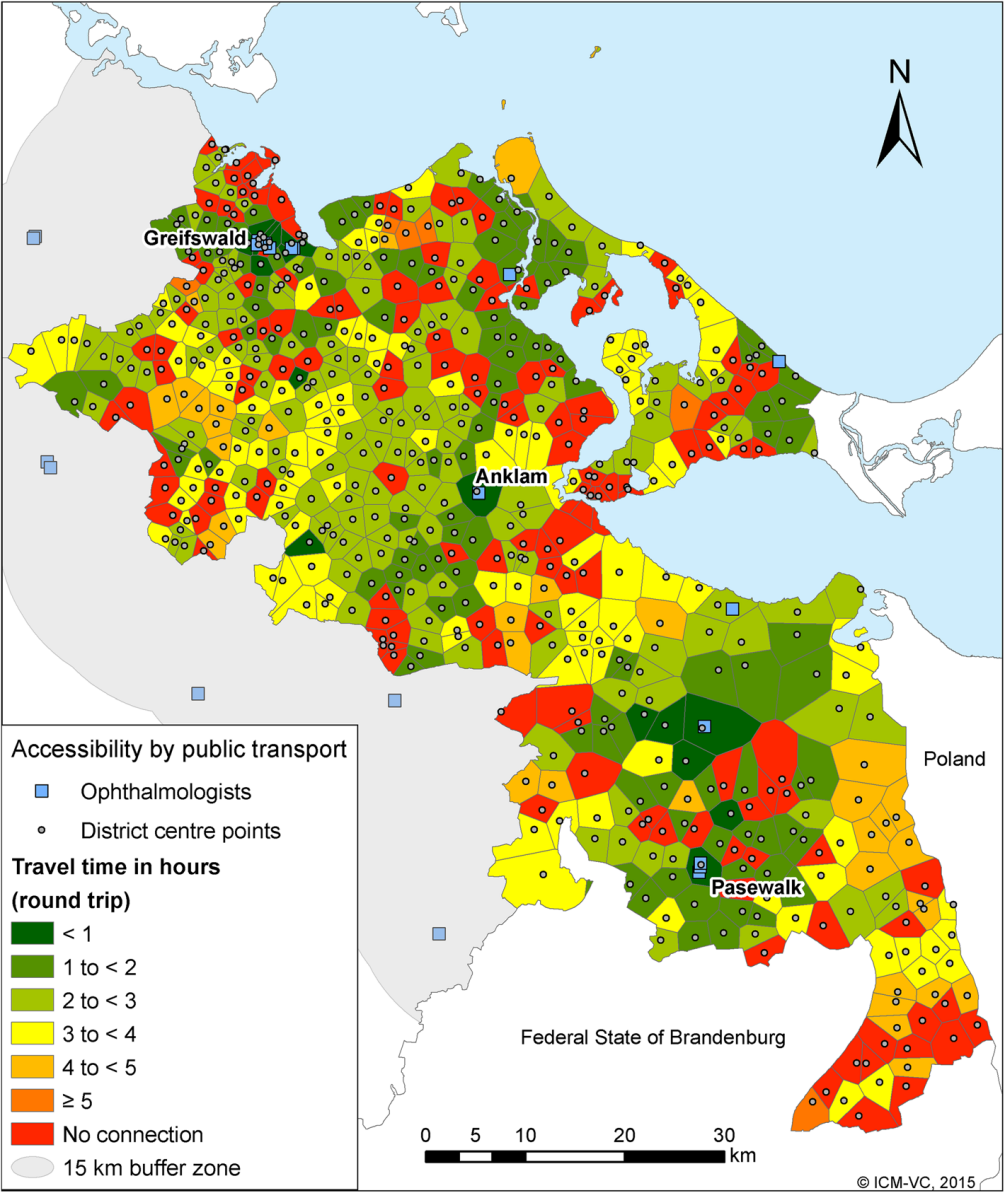
Accessibility of ophthalmologists by public transport

The results of the accessibility by public transport show the total travel time-round trip-in hours (Figs. 3 and 4). In contrast to the accessibility by car, the distance between starting and end point does not always correspond to the travel time. 68.2 % of the inhabitants have travel times up to 1 h to the next GP (round trip); 82.5 % up to 2 h; 93.7 % up to 3 h and 3.8 % have no connection within 1 day under our model assumptions (Table 3). Only 36.2 % of the inhabitants have travel times up to 1 h (round trip) to the next ophthalmologist. 4.6 % inhabitants have no connection within 1 day. 37.4 % respectively 40.0 % of the inhabitants have travel times up to 1 h to the nearest internists and urologist; 6.5 % and 7.1 % inhabitants, respectively, have no connection to these specialists. It takes on average 1 h and 39 min to reach the next GP and get back home with public transport, whereas the average travel time to the three selected specialist physicians is more than 2 h (ophthalmologist: 2 h and 9 min; specialist internist: 2 h and 23 min; urologist: 2 h and 39 min) (Table 4).

**Table 3:** Accessibility of GP by public transport (round trip) with the proportion of affected districts and inhabitants

Time (Hours)	Number of districts	Number of inhabitants	Proportion of districts	Proportion of inhabitants
< 1	53	163,062	11	68
1 to < 2	152	34,284	33	14
2 to < 3	119	26,753	26	11
3 to < 4	25	5,085	5	2
4 to < 5	6	911	1	0
≥ 5	1	34	0	0
No connection	109	8,973	23	4
Total	464	239,102	99^a^	99^a^

^a^The sum of the percentages is 99 % because of rounding

**Table 4:** Travel times (in hours and minutes) by public transport to GP, ophthalmologists, specialist internists and urologists

Physicians	Mean	Standard deviation	Maximum
General practitioners	1 h 39 min	1 h 03 min	5 h 10 min
Specialist internists	2 h 23 min	1 h 40 min	6 h 51 min
Ophthalmologists	2 h 09 min	1 h 16 min	6 h 30 min
Urologists	2 h 39 min	1 h 50 min	7 h 17 min

The differences in mean travel time by public transport between the physician groups are significant (*p* < .0001). The Tukey correction showed here that all groups differ significantly with respect to each other except for specialist internists and ophthalmologists.

## Discussion

The accessibility by car can be calculated under more realistic conditions and is more robust against changes in the assumptions whereas the assessment of the accessibility by public transport depends more on the model. The approach of Lovett et al. works in the situation, but requires specific simplifications and estimations. Exact locations of bus stops, several connecting buses and services provided only on certain weekdays were not considered. Instead of exact locations closest nodes on the road network to each postcode with residents and to the nearest GP surgery were the origins for Lovett’s analysis [16]. In our study center points of the districts, bus and train stops and the practice locations were determined. The bus stops were actively validated and their coordinates manually assigned using handheld GPS devices. A more detailed origin-location-basis than district central points would be desirable, but leads to a considerably larger data requirement that would be very computation-intensive.

The calculation of Liu and Zhu used walking and travel times in an urban setting with regular time schedules. Individual destinations and schedules of public transport were not taken into account, which makes the Accessibility Analyst more suitable for large scale transportation analysis and planning. The Accessibility Analyst allows the evaluation of the accessibility of places in general rather than on an individual level [15]. The purpose of our study was to analyze the accessibility of the selected physicians based on the people in the study region. A strength of our study is that the calculation is based on the original schedules of all regional bus and train lines. Hence, rather than model estimations real time schedules were used.

Benenson et al. analyzed accessibility regarding direct trips and trips with one transfer. The “Urban.Access”-tool is meant for urban regions. Destinations with more than one transfer were not considered. The limitation of the number of transfers caused large accessibility gaps in specific situations [13]. The model used in our study considered walking distances to origins and to destinations and walking distances between transfers as well as waiting times. The transfer times between lines or transport modes were determined specifically for each connection rather than averaged or assumed to be constant in all transfers. Hence, waiting times reflect the actual time tables.

Tribby and Zandbergen produced realistic results with their multimodal network model. They assessed time savings from the addition of two rapid buses in the considered study region [18]. Also the study of Djurhuus et al. was well suited to assess accessibility [14]. The advanced accessibility model of Salonen and Toivonen revealed more realistic results than the simple and intermediate models. But the advanced model was very data and computing-time intensive [17]. A multimodal network is promising, but public transport data often lacks the required structure and availability. Tribby’s and Zandbergen’s study region is relatively small in its dimensions as well as in its variety of different transport companies and transport systems. Djurhuus et al. could use one data source with uniformly structured data. A multimodal network in ArcGIS is more feasible for relatively small regions and for data with a more uniform structure.

Despite high-resolution input data, this assessment of the accessibility by car and by public transport is based on models that include a range of assumptions. Both modes use the assumption that patients consult the closest physician. However, in real life the decision for a physician may involve other reasons than just distance, like commuter relations and physician reputation [26, 27]. The assessment of the accessibility by public transport implies further assumptions. The definition of the walking distances is somewhat arbitrary. If in practice the distances between the district central points and the bus or train stops or the medical facilities were larger than determined, then additional connections may exist. Bus/train stops further away than 1000 m from district central points or practices can cause both shorter and longer travel times depending on the specific case. Also the determination of the earliest starting time, the assumed duration of the appointment in the doctor’s practice or the determination of the pedestrian speed are assumptions. Walking a little faster can allow to reach another bus within a defined time window. However, since the focus of this work is on elderly people, we chose distances and a walking speed that seem plausible for this group.

Travel times highly depend on the weekday and daytime. In urban settings rush hours and traffic jams may alter travel time. In this sparsely populated region traffic jams and rush hours are only of minor importance. However, in rural regions public transport is focused on school traffic [23]. As a consequence, travel time fundamentally depends on school versus vacation times, the time, when school starts in the morning and the times when it ends in the afternoon. Therefore, the most favorable conditions for the model were chosen: the doctor’s appointment at Tuesday 11 am during school time. All other conditions yielded lesser accessibilities. In summary, all assumptions were oriented to realistic or optimistic conditions and the consideration of all transportation modes (including transfers) and time schedules allowed a high degree of accuracy.

A cross-sectional survey in the region Vorpommern assessed the satisfaction of people ≥ 60 years with the GP-accessibility. In total 81 % were satisfied, 19 % were not satisfied with the accessibility of the GP [12]. Women were significantly less satisfied (22 %) than men (15 %). The satisfaction was significantly lower in rural-peripheral regions compared to urban regions. Satisfaction was worse with longer travel times. [12]. These results correspond to the findings of our analysis.

Long travel times and a bad accessibility of physicians may have effects on the utilization of health care providers and in the consequence on the health situation of patients with limited mobility in rural regions. Further research is needed to examine possible effects of long travel times on health outcomes.

Medical planning in Germany bases on the ratio of inhabitants per doctor for 14 different medical specialist groups and 9 different region types (types of settlements of the Federal Institute for Building, Urban Affairs and Spatial Development (BBSR)) [28, 29]. In 2010 a demographic factor has been introduced for GPs. Accessibility and travel times are not considered in the German medical planning yet. But the results of this study show that medical planning should not be detached from accessibility, planning of infrastructure and logistics.

## Conclusions

We examined the accessibility of different groups of physicians in a rural area with a low population density, both by car and by public transport. On average, travel times to GPs are lower than to any of the medical specialists. Travel times in the public transport system are high for a substantial part of the population and some villages have no connection to a physician practice. About 8,000 to 15,000 people have no access to the examined physicians with public transport (3 to 6 % of the population in the study region).

The low population density in rural regions limits the cost-efficient operation of public transport. Concepts that consider the planning of medical care and public transport simultaneously are needed. Adequate medical care should be evaluated not only based on availability but also by accessibility of medical facilities [30].

## Abbreviations

BBSR: Federal Institute for Building, Urban Affairs and Spatial Development
GIS: Geographic Information System
GP: General Practitioner
MV: Mecklenburg-Vorpommern
VG: Vorpommern-Greifswald
